# Pruritus is a common feature in sheep infected with the BSE agent

**DOI:** 10.1186/1746-6148-4-16

**Published:** 2008-04-29

**Authors:** Timm Konold, Gemma Bone, Alberto Vidal-Diez, Raul Tortosa, Andrew Davis, Glenda Dexter, Peter Hill, Martin Jeffrey, Marion M Simmons, Melanie J Chaplin, Susan J Bellworthy, Christine Berthelin-Baker

**Affiliations:** 1 Veterinary Laboratories Agency Weybridge, Woodham Lane, Addlestone, UK; 2 Royal Veterinary College, Population Biology and Disease Control Research Group, North Mymms, Hatfield, UK; 3 Department of Animal Medicine and Surgery, Institute of Neuroscience, Veterinary Faculty, Universitat Autònoma de Barcelona, Barcelona, Spain; 4 Pathology, Infectious Disease & Biosecurity, School of Veterinary Science, University of Queensland, Brisbane, Australia; 5 ADAS Drayton, Alcester Road, Stratford upon Avon, UK; 6 Veterinary Laboratories Agency Lasswade, Pentlands Science Park, Bush Loan, Penicuik, UK; 7 All Animals Neurology & Neurosurgery, Atlanta, Georgia, USA

## Abstract

**Background:**

The variability in the clinical or pathological presentation of transmissible spongiform encephalopathies (TSEs) in sheep, such as scrapie and bovine spongiform encephalopathy (BSE), has been attributed to prion protein genotype, strain, breed, clinical duration, dose, route and type of inoculum and the age at infection. The study aimed to describe the clinical signs in sheep infected with the BSE agent throughout its clinical course to determine whether the clinical signs were as variable as described for classical scrapie in sheep. The clinical signs were compared to BSE-negative sheep to assess if disease-specific clinical markers exist.

**Results:**

Forty-seven (34%) of 139 sheep, which comprised 123 challenged sheep and 16 undosed controls, were positive for BSE. Affected sheep belonged to five different breeds and three different genotypes (ARQ/ARQ, VRQ/VRQ and AHQ/AHQ). None of the controls or BSE exposed sheep with ARR alleles were positive. Pruritus was present in 41 (87%) BSE positive sheep; the remaining six were judged to be pre-clinically infected. Testing of the response to scratching along the dorsum of a sheep proved to be a good indicator of clinical disease with a test sensitivity of 85% and specificity of 98% and usually coincided with weight loss. Clinical signs that were displayed significantly earlier in BSE positive cases compared to negative cases were behavioural changes, pruritic behaviour, a positive scratch test, alopecia, skin lesions, teeth grinding, tremor, ataxia, loss of weight and loss of body condition. The frequency and severity of each specific clinical sign usually increased with the progression of disease over a period of 16–20 weeks.

**Conclusion:**

Our results suggest that BSE in sheep presents with relatively uniform clinical signs, with pruritus of increased severity and abnormalities in behaviour or movement as the disease progressed. Based on the studied sheep, these clinical features appear to be independent of breed, affected genotype, dose, route of inoculation and whether BSE was passed into sheep from cattle or from other sheep, suggesting that the clinical phenotype of BSE is influenced by the TSE strain more than by other factors. The clinical phenotype of BSE in the genotypes and breed studied was indistinguishable from that described for classical scrapie cases.

## Background

After the discovery that bovine spongiform encephalopathy (BSE) and variant Creutzfeldt-Jakob disease (v-CJD) were caused by the same agent [1] research on transmissible spongiform encephalopathies (TSEs) targeted other farm animal species that may harbour the BSE agent. Sheep were a particular concern because scrapie, another TSE, has been endemic in sheep flocks for more than 200 years, and if sheep had been fed BSE infected meat and bone meal, there would have been the possibility that it could go undetected if mistaken for scrapie. Several studies have been conducted at the Veterinary Laboratories Agency (VLA) since 1997 to investigate the clinical presentation, pathology, pathogenesis, diagnosis and transmission of BSE in sheep.

As for scrapie, the susceptibility of sheep to BSE is largely determined by polymorphisms (principally at codons 136, 154 and 171) of the gene that encodes the prion protein (PrP). Sheep most susceptible are homozygous for alanine at codon 136, glutamine at codon 171, and either arginine (ARQ/ARQ) or histidine (AHQ/AHQ) at codon 154 [2], whereas arginine at codon 171 (ARR) is associated with increased resistance [3]. Although intracerebral infection of ARR/ARR sheep with BSE has produced clinical disease [4], it is not known whether oral infection can produce clinical disease in this genotype within the natural life span of sheep. A more recent study proposed that other polymorphisms at codons 137, 142, 168 and 176 of the PrP gene may also influence the susceptibility of sheep to experimental BSE [5,6].

Despite considerable variation in the clinical signs of scrapie in sheep, three main types have been described, a pruritic form, a paralytic form without pruritic behaviour, and an atypical form, which usually includes signs of a cerebellar disease [7-11]. It is largely unknown which factors determine the clinical presentation but it may be the result of differences between strains [12,13], genotypes [14] or clinical duration [14,15]. Other factors, such as breed, dose or age at infection may also be contributory; for example, differences in the vacuolar lesion profiles in scrapie have been attributed partly to the sheep breed [16].

The first study conducted in the UK described BSE in sheep as an acute disease with a duration of less than one week regardless of the route of inoculation (intracerebral or oral), which was characterised by ataxia resulting in recumbency; pruritus was usually absent [17,18]. This was subsequently confirmed in an oral transmission study [2]. By contrast, intraperitoneal infection of a sheep with BSE resulted in a protracted clinical course and at the late-stage intense pruritus and ataxia [19], and in a Dutch study four orally inoculated sheep developed clinical signs that included ataxia, head tremor and a nibble reflex [20]. A more detailed clinical study of 28 sheep inoculated intracerebrally or intravenously with BSE suggested that BSE is "more likely to present with sudden onset of ataxia in the absence of pruritus compared to scrapie although 42% presented with pruritus alone and 25% displayed pruritus in combination with ataxia" [21]. Observed variations were attributed to the different routes of inoculation, different doses and different inocula (BSE brain or blood from BSE-affected sheep).

The objective of this paper is to describe the clinical signs of BSE in sheep, throughout its clinical course and at end-stage. The bulk of the data concerns ARQ homozygous Suffolk and Romney sheep, which were orally challenged with the BSE agent or acquired the infection naturally in an experimental flock. A small number of other breeds and genotypes and two sheep inoculated intracerebrally were also studied. To evaluate whether disease-associated clinical markers exist, the clinical features were compared with those of culled sheep of different genotypes and breeds that had not been challenged with BSE (cohort controls) or had been challenged but not confirmed pathologically as BSE cases. For the purpose of this paper, a BSE case is an animal that developed undisputable clinical features of BSE and was positive on postmortem examination of the brain.

## Results

Forty-seven sheep (34%) were positive for BSE by postmortem tests on brain tissue. Eleven of these were culled at pre-determined time points to study BSE pathogenesis and tissue distribution. The numbers of BSE cases in sheep of each genotype and breed and the doses, routes of infection and incubation periods are detailed in Table 1. Table 2 lists the details of unchallenged control sheep and challenged sheep without pathological confirmation of BSE.

**Table 1:** Genotype, breed distribution with inoculation details and incubation periods of confirmed BSE cases

**Genotype**	**Breed**	**Dose (at age)**	**n**	**IP**
AHQ/AHQ	Cheviot	5 g orally (4–9 months)	4	17, 17, 18, 18
ARQ/ARQ	Poll	5 g orally (6 months)	2	22, 29
	Dorset	0.5 g orally (6 months)	1	25
	Romney	5 g orally (4–7 months)	17	29 (2.1)
		0.5 g orally (6 months)	2	23, 24
		"Natural infection" (experimental flock)	1	24
	Suffolk	5 g orally (4–8 months)	14	24 (0.9)
		0.5 g orally (6 months)	1	25
		"Natural infection" (experimental flock)	1	21
		1 ml intracerebrally (8 months)	2	17, 18
VRQ/VRQ	Cheviot	5 g orally (4 months)	2	51, 54

IP = Incubation period in months (always rounded down), defined as time period from inoculation (birth for naturally infected animals) to cull. The mean incubation period with the standard error in parentheses is given if the numbers exceed four. Excluded are the scheduled culls (seven Romney, four Suffolk sheep) in the oral pathogenesis studies (e.g. the pre-clinical cases).

**Table 2:** Genotype, breed distribution with inoculation details and incubation periods of BSE-negative sheep

	**Genotype**	Breed	**n**	**Oral dose (at age)**	**IP**
**Unchallenged (controls)**	ARQ/ARQ	Romney	3	None	15, 21, 34
		Suffolk	2	None	3, 15
	ARQ/ARR	Romney	4	None	10, 21, 33, 45
	ARR/ARR	Romney	4	None	9, 21, 33, 45
		Suffolk	3	None	9, 34, 45
	
	**TOTAL**		**16**		

**Challenged**	ARQ/ARQ	Romney	5	5 g (6 months)	12 (1.22)
		Suffolk	7	5 g (4 months)	9 (2.4)
		Suffolk	1	0.5 g (6 months)	38
	ARQ/ARR	Romney	17	5 g (6 months)	41 (6.7)
		Romney	1	0.05 g (6 months)	50
		Suffolk	13	5 g (4 months)	32 (6.1)
	ARR/ARR	Romney	17	5 g (6 months)	42 (7.3)
		Suffolk	15	5 g (4 months)	29 (3.4)
	
	**TOTAL**		**76**		

IP = Incubation period in months (always rounded down), defined as time period from inoculation to cull and – for unchallenged animals in the oral BSE pathogenesis study – from test group exposure and cull. The mean incubation period with the standard error in parentheses is given if the numbers exceed four.

### Clinical assessment prior to cull

Table 3 compares selected clinical signs observed in BSE-positive and negative sheep up to four weeks prior to cull. Behaviour, such as yawning, flehmen, snorting and sneezing, was displayed too infrequently to be used as a clinical marker.

**Table 3:** Selected clinical signs displayed up to 4 weeks prior to cull

**Postmortem diagnosis**	**BSE POSITIVE**		**BSE NEGATIVE**		***P***
Clinical sign	Present	Absent	Present	Absent	*

*Behavioural abnormalities*					

Change in behaviour (dull or nervous)	27 (61%)	17 (39%)	7 (10%)	60 (90%)	+++
Bruxism when handled	11 (27%)	30 (73%)	4 (6%)	58 (94%)	+
Bruxism when free during examination	6 (15%)	35 (85%)	4 (6%)	58 (94%)	ns
Bruxism frequently during observations	7 (23%)	12 (40%)	0 (0%)	58 (89%)	+++
Bruxism occasionally during observations	6 (20%)		0 (0%)		+++
Bruxism during observations once	5 (17%)		7 (11%)		ns

*Sensory abnormalities (pruritus)*					

Rubbing or scratching frequently	19 (63%)	2 (7%)	8 (12%)	27 (43%)	+++
Rubbing or scratching occasionally	7 (23%)		11 (17%)		+
Rubbing or scratching once	2 (7%)		19 (28%)		ns
Nibbling frequently	5 (17%)	13 (43%)	0 (0%)	43 (68%)	+
Nibbling occasionally	7 (23%)		0 (0%)		++
Nibbling once	5 (17%)		20 (32%)		ns
Positive scratch test	35 (85%)	6 (16%)	1 (2%)	61 (98%)	+++
Fleece changes	19 (40%)	28 (60%)	10 (11%)	77 (89%)	++
Alopecia	26 (55%)	21 (45%)	3 (3%)	86 (97%)	+++
Skin lesions	15 (32%)	32 (68%)	3 (3%)	86 (97%)	+++
Self-induced scratch response	27 (75%)	9 (25%)	3 (5%)	62 (95%)	+++

*Abnormalities in movement*					

Tremors	22 (52%)	20 (48%)	4 (6%)	64 (94%)	+++
Ataxia	23 (49%)	24 (51%)	1 (1%)	90 (99%)	+++

*Physical changes*					

Weight loss >10%	12 (25%)	9 (22%)	1 (2%)	48 (70%)	+++
Weight loss 5–10%	5 (13%)		1 (2%)		+
Weight loss < 5%	15 (35%)		7 (10%)		+++
Weight remained the same	2 (5%)		11 (16%)		ns
Loss of body condition	20 (57%)	15 (43%)	13 (34%)	25 (66%)	ns

* *P*-values: + ≤ 0.01;

++ ≤ 0.001;

+++ ≤ 0.0001;

ns statistically not significant (P > 0.05)

The BSE-negative subset included challenged and unchallenged animals. The homogeneity of the clinical signs within this subset was proven by Fisher's exact test (*P *> 0.05)

Pruritus, defined as the occurrence of a positive scratch test (see Additional files 1 and 2: movies 1 and 2 showing sheep with a positive scratch test) or – if this was not tested – a self-induced scratch response (see Additional file 3: movie 3), occasional or frequent rubbing, scratching or nibbling (see Additional file 4: movie 4 showing two sheep with pruritic behaviour), was present in 38 (86%) out of 44 BSE positive sheep. In advanced cases in ARQ homozygotes, a clear nibbling response could sometimes be elicited by merely touching the sheep (see Additional file 5: movie 5 of a sheep with a strong scratch response on touch), whereas nibbling was less pronounced or required more forceful scratching in the AHQ homozygous Cheviots (see Additional file 6: movie 6 showing a sheep that exhibits a more evident response to scratching when not restrained).

The clinical signs displayed by the six non-pruritic BSE cases, which were culled at pre-determined time-points, are shown in Table 4.

**Table 4:** Clinical findings in non-pruritic BSE cases of the oral BSE pathogenesis study

**Animal**	**SM1391**	**SM1430**	**SM1394**	**SP3652**	**SM1435**	**SM1404**
**Breed and genotype**	Romney ARQ/ARQ	Romney ARQ/ARQ	Romney ARQ/ARQ	Suffolk ARQ/ARQ	Romney ARQ/ARQ	Romney ARQ/ARQ
**MPI**	16	16	22	16	22	22
**Postmortem test result**	HP neg. IHC pos.	HP neg. IHC pos.	HP neg. IHC pos.	HP neg. IHC pos.	HP pos. IHC pos.	HP pos. IHC pos.
**Behaviour change**	No	No	No	No	N/A	No
**Pruritic behaviour**^1^	No	No	Rubbing once	No	Rubbing once	No
**Fleece change**	No	No	No	No	No	No
**Skin Lesion**	No	No	No	No	No	No
**Bruxism when observed or examined**	No	No	No	No	No	No
**Tremors**	No	No	No	No	N/A	No
**Ataxia**	No	No	No	No	No	No
**Weight loss**	No	<5%	No	No	<5%	<5%

^1 ^The display of a positive scratch test, a self-induced scratch response, self-nibbling, rubbing or scratching

HP = Histopathological examination of the brain

IHC = Immunohistochemical examination of the brain

neg./pos. = negative/positive

N/A = not assessed

Romney sheep were inoculated at approximately six months of age, Suffolk sheep at approximately four months of age.

Based on the criteria that a positive scratch test was an indication of BSE infection in the brain (by means of PrP^d ^detection), the test sensitivity was 84% and test specificity 98% (Positive Predictive Value = 97%, Negative Predictive Value = 91%, data see Table 3). Of the six cases that were positive on postmortem tests without displaying a positive scratch test (ARQ/ARQ Suffolk and Romney sheep), three were culled at 16 months post inoculation (mpi) and displayed no specific signs (SM1391, SM1430, SP3652, see Table 4) whilst the other three animals were killed between 21 and 22 mpi and displayed pruritic behaviour, such as frequent or occasional rubbing and nibbling in combination with alopecia in two and a self-induced nibble reflex in one animal. One sheep with a positive scratch test on two consecutive examinations that had no detectable PrP^d ^in the brain and lymphoreticular tissue was a dosed ARQ/ARQ Suffolk culled at 16 mpi, which did not display any other clinical signs except for one event of rubbing. Other pruritic behaviour (a self-induced nibble reflex, occasional or frequent rubbing, scratching or nibbling) was observed in 19 (29%) of 65 BSE-negative sheep (54 dosed sheep, 11 undosed controls) that were observed sufficiently within four weeks prior to cull. The genotype of these sheep was either ARQ/ARR (n = 6) or ARR/ARR (n = 13); all were dosed with the BSE agent and culled between 21 and 89 mpi (mean 41 mpi, SEM 3).

All confirmed BSE cases that were ataxic prior to cull (see Additional file 7: movie 7 of a sheep with mild proprioceptive deficits and hind limb ataxia) also displayed pruritus, characterised by either a positive scratch test (see Additional file 5: movie 5 showing the same animal with a positive scratch test) or – if this was not tested (six animals) – by frequent rubbing, scratching and nibbling.

The combination of changed behaviour, pruritus and ataxia was displayed by 17 (40%) of 43 BSE positive cases that had each sign assessed but was not observed in BSE-negative sheep.

Over-reactivity to auditory stimuli, such as repeated flinching in response to a hand clap, was displayed by three (7%) of 42 tested BSE-affected sheep and none of the BSE-negative sheep. The assessment of cranial nerve function was unremarkable in most cases except for either unilateral or bilateral decreases in the menace response in three (8%) of 39 BSE-affected sheep (not observed in any of the BSE negative culls). Sheep of all groups (BSE positive and BSE negative including unchallenged controls), particularly Romney sheep, often exhibited a weak pupillary light response. As visual deficits were not evident and no eye abnormalities were apparent by indirect ophthalmoscopy, this finding was considered to be normal.

#### Intercurrent diseases

Six sheep with at least one ARR allele were culled with intercurrent diseases and had a negative BSE test result on brain and lymphoreticular tissue.

A Romney wether (SR6128, ARQ/ARR, orally dosed with 0.05 g of BSE brainstem at six months of age and culled at 50 mpi) presented with progressive weight loss, bruxism (teeth grinding) during handling and alopecia on the bridge of the nose. Increased serum liver enzyme activity was suggestive of a liver disease; hepatitis was diagnosed histopathologically.

A Suffolk wether (SP3611, ARQ/ARR, orally dosed with 5 g of BSE brainstem at five months of age and culled at 90 mpi) developed a head tremor at rest. This sheep had a stiff, mildly ataxic gait and responded to scratching of the dorsum with lip tremor and head tilt (inconclusive scratch test). Haematology and blood biochemistry were unremarkable. Degenerative osteochondritis of the femoro-tibial joints and acute oedema of the brain suggestive of enterotoxaemia following clostridial replication was diagnosed, although there was no clinical and pathological evidence of enteritis.

Another four sheep had displayed episodes of lateral recumbency with paddling of the limbs, which were described as "fits" by animal husbandry staff and may have been seizures.

A Romney ewe (SM1408, ARQ/ARR, orally dosed with 5 g of BSE brainstem at six months of age and culled at 85 mpi) was found recumbent in the pen paddling with its limbs; the sheep had been unremarkable at a neurological examination a few days earlier. An adenoma of the pituitary gland was diagnosed.

A Romney ewe (SM1453, ARQ/ARR, orally dosed with 5 g of BSE brainstem at six months of age, culled at 91 mpi) had difficulty standing for 15 minutes after a sudden fit but had been clinically unremarkable before. A leucoencephalopathy of undetermined but possible metabolic origin and an abscess at the base of the tongue were found.

A Romney ewe (SM1418, ARR/ARR, orally dosed with 5 g of BSE brainstem at six months of age, culled at 97 mpi) presented with weight loss, bruxism, head tremor and had a history of fits. A diagnosis of leucoencephalopathy, possibly as a result of a metabolic disease, was made.

A Romney wether (SM1450, ARQ/ARR, orally dosed with 5 g of BSE brainstem at six months of age, culled at 104 mpi) had a history of occasional fits since 88 mpi; these progressively became more frequent. In addition, the sheep developed a left corneal oedema with ulceration but no other clinical signs were seen. A leucoencephalopathy, similar to the previous two cases, was diagnosed.

### Clinical progression

Table 5 displays the results of the survival analysis for 60 animals, including 23 BSE-positive sheep, which were monitored clinically from approximately 13 mpi until cull.

**Table 5:** Survival analysis test to compare the times of the first appearance of a particular clinical sign in BSE positive and negative cases.

	**Presence during the monitoring period**		*P*-value *	
**Clinical sign**	**Negative cases (n)**	**Positive cases (n)**	**Log-rank test**	**Wilcoxon test**
Behaviour change	17	16	++	
Rubbing/scratching	34	23	ns	
Nibbling	25	21	+	
Positive scratch test	4	18	+++	
Fleece change	28	18		ns
Alopecia	4	14	+++	
Skin lesions	5	10	+	
Self-induced scratch response	7	18	+++	
Bruxism when handled	9	9	+++	
Bruxism when free during examination	7	5		ns
Bruxism at observation	17	17	+	
Tremors	2	14	+++	
Ataxia	2	13		+++
Weight loss	29	22		+
Loss of body condition	20	18	+++	

* *P*-values: + ≤ 0.04;

++ ≤ 0.001;

+++ ≤ 0.0001;

ns statistically not significant (P > 0.05)

The analysis was compromised by the low number of cases, in particular those that had no detectable PrP^d ^in the brain, which displayed some of the clinical signs used for comparison, such as tremor or ataxia. BSE positive cases displayed most signs significantly earlier than BSE-negative sheep, which included six undosed controls. Only the first display of fleece changes and bruxism during the examination when the animal was undisturbed (bruxism when free) was not significantly different (see Additional file 8: plots, which displays the plots of Kaplan-Meier estimates for the first appearance of each clinical sign).

**Figure 1:**
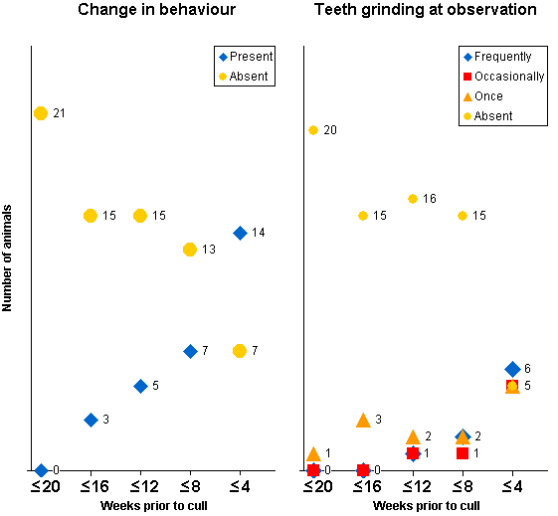
**Behavioural signs displayed in 21 BSE cases observed over a period of 20 weeks prior to being culled because of disease progression**. The total number of animals per time period differed because not every sign was assessed during each period.

**Figure 2:**
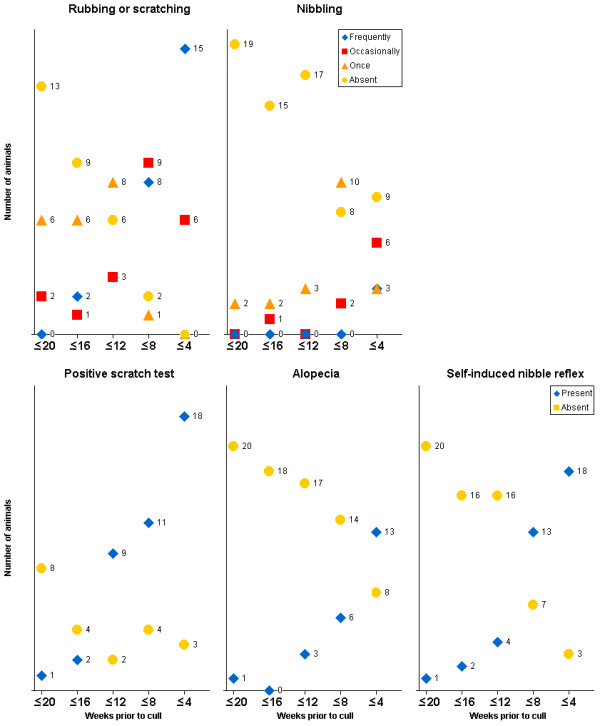
**Signs of pruritus displayed in 21 BSE cases observed over a period of 20 weeks prior to being culled because of disease progression**. The total number of animals per time period differed because not every sign was assessed during each period.

**Figure 3:**
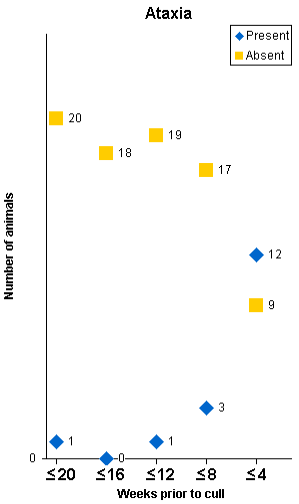
**Signs of ataxia displayed in 21 BSE cases observed over a period of 20 weeks prior to being culled because of disease progression**. The total number of animals per time period differed because not every sign was assessed at each time.

**Figure 4:**
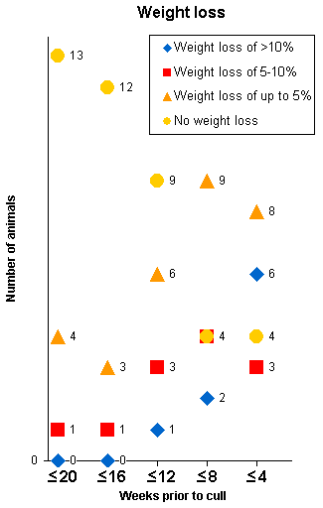
**Unspecific signs displayed in 21 BSE cases observed over a period of 20 weeks prior to being culled because of disease progression**. The total number of animals per time period differed because not every sign was assessed at each time.

The clinical signs in 21 clinical suspects with confirmed BSE that were monitored regularly from at least 20 weeks prior to cull are displayed in Figures 1, 2, 3, 4. Signs usually progressed over a period of 20 weeks. Individual clinical signs were usually displayed in single animals only or expressed mildly (rubbing/scratching, nibbling and weight loss) at 16 to 20 weeks prior to cull but became more frequent or severe as the disease progressed.

## Discussion

This study demonstrated that pruritus is a common feature in clinical cases of ovine BSE. Forty-one (87%) of the 47 BSE-positive sheep displayed signs of pruritus. A repeatable stereotypical response (nibbling or head/body movements) to the scratch test, which has been associated with pruritus in sheep with scrapie [14], was elicited in 85% of 41 BSE-positive sheep.

The six non-pruritic BSE cases, which were culled at pre-determined time points, displayed no specific signs of neurological disease; three had minor loss of weight prior to cull. Although the occurrence of even minor weight loss was significantly greater in clinical BSE cases (see Table 3), this sign is not specific for BSE and was also observed in BSE-negative sheep at some stage during their life. Thus, we concluded that these six sheep represented pre-clinical BSE cases. All six sheep were culled between 16 and 22 mpi, and PrP^d ^accumulation was detected by immunohistochemical examination of parts of the central nervous system (CNS) [22]; three did not present with disease-specific vacuolation in the brain. As most previous studies of experimental TSE in sheep do not include detailed clinical assessments, it is difficult to know how frequently pre-clinical cases may be detected by postmortem brain tests. In one study, orally dosed sheep with scrapie displayed early clinical signs around the same time as sparse PrP^d ^accumulation was detectable in the CNS whereas one naturally infected sheep displayed no clinical signs and presented with vacuolation and PrP^d ^accumulation in the brain [23], similarly to our three non-pruritic BSE cases.

One sheep displayed a positive scratch test on two consecutive examinations prior to cull, which was highly suggestive of BSE, even though PrP^d ^was not detected in the brain or lymphoreticular tissues of this sheep. Aside from TSEs, a positive scratch response may be elicited in animals with pruritic skin conditions, such as psoroptic mange [24,25]. This sheep showed no significant pruritic behaviour, alopecia or skin lesions indicative of pruritus. Additionally, searches for ectoparasites remained negative in several "suspect" sheep tested at various time points throughout the studies, suggesting that our sheep remained ectoparasite free. Other pruritic behaviour (a self-induced nibble reflex, occasional or frequent rubbing, scratching or nibbling) was also observed in 19 challenged sheep with at least one ARR allele, which were not confirmed as BSE cases by postmortem tests. Clinical and neurological signs have been reported in experimentally challenged ARR/ARR sheep with detectable PrP^d ^in the brain [4]. If the pruritic behaviour observed in our sheep was indeed caused by BSE we would have expected that the disease was confirmed by postmortem tests. A clinical diagnosis of TSE is more likely to be made if unequivocal neurological signs, such as head tremor or ataxia are present. In our studies, several sheep developed skin lesions that led to culling for welfare reasons at a time point preceding the onset of ataxia in their cohort challenged group. The severity of the skin lesions may not always reflect disease progression, given the influence of other factors, such as the presence of hard sharp surfaces in the pen and the sheep's individual propensity to rub or self mutilate. In addition, clinical progression could not be monitored in the eleven BSE cases that were culled at pre-determined time points to study BSE pathogenesis and tissue distribution. However, the clinical signs displayed by these sheep up to their cull date were identical to those noted in their cohort challenge group, contributing to the establishment of the clinical phenotype of BSE in each challenge group.

Six sheep inoculated with the BSE agent were culled due to intercurrent diseases, five of which displayed neurological signs without pruritus. All of these six sheep were either homozygous or heterozygous R at codon 171, and postmortem tests showed no evidence of BSE, as assessed by the absence PrP^d ^in the brain and lymphoreticular tissue of these sheep, and none of the sheep in our studies with this genotype have so far had BSE confirmed by postmortem tests. Three sheep with histopathological evidence of leucoencephalopathy of undetermined origin presented with episodes resembling seizures although loss of consciousness, salivation, urination or defecation, which usually accompany seizures [26], were not described. Seizures are most frequently associated with focal or diffuse forebrain or thalamic lesions caused by infections, toxins, tumours, nutritional deficiencies or metabolic diseases [27]. A blood sample was not examined for the presence of metabolic disease (e.g. hypomagnesaemia, thiamine deficiency) but this as well as toxins were considered unlikely because of the strict feeding and housing regime used in our studies.

Our findings that sheep with BSE are generally pruritic whereas ataxia is displayed later in the clinical course are in contrast to the observations of others [17,21]. It is possible that the route of inoculation (oral dosing or natural transmission in our experimental flocks) results in a different clinical presentation compared to parenteral inoculation mostly used by others. Pruritus was not observed in a limited number of BSE cases described in previous studies, but some of the sheep were kept at pasture and the methods by which clinical signs were assessed or recorded were not detailed [17,18]. The very detailed clinical monitoring in our studies likely contributed to the detection of signs of pruritus, which could have been overlooked in another setting. During our prolonged passive observations, we noted that the sheep would initially "freeze" and just stare at the observer, and that they would return to their "normal" group activities only after the first 10–20 minutes of observation. This "freezing behaviour" explains why pruritus was seldom witnessed during the shorter daily or routine husbandry procedures, even in cases that had displayed pruritus for some time during passive observation sessions. Continuous video surveillance may be a good alternative to prolonged observations, since it has been shown that initial signs of pruritus were detected by video surveillance but were not apparent to observers [28]. Another useful contribution to the detection of signs of pruritus was the systematic assessment of the scratch test, which was not used in previously reported studies of BSE in sheep. One BSE-affected sheep, which presented with a positive scratch test, tremor and changed behaviour (initially nervousness and later dullness), did not start pruritic activity until 20 minutes into an observation session, indicating that episodes of pruritic activity can be missed if sheep are observed for only a short time. Pruritic activity may be increased in association with resting behaviour in sheep. Disturbances caused by noise or the presence of humans result in a decrease in resting behaviour and an increase in movement and head alert reactions in sheep [29], likely more so in nervous sheep.

When the presence of each individual clinical sign was compared between BSE-positive and negative cases over the monitoring period from approximately 13 mpi until cull, 13 of 15 signs appeared significantly earlier (*P *< 0.05) in BSE positive cases. For example, all positive cases with observed bruxism during handling displayed this sign before 122 weeks post inoculation (p.i.) whilst approximately 89% of the BSE-negative cases presented with this sign after 133 weeks. Only fleece changes and bruxism during the examination when the sheep was undisturbed (bruxism when free) were not displayed earlier in positive or negative cases. Both are very unspecific signs and may occur in "normal" animals. In addition, every statistical analysis is dependent on the number of observations. A low number of observations, as seen for "bruxism when free", results in wide confidence intervals or prevents meaningful statistical analysis, which is why the degree of rubbing or nibbling was also not taken into consideration for the survival analysis, although frequent rubbing or nibbling was rarely or never observed in BSE-negative cases (exposed sheep that were negative for PrP^d ^or unexposed controls) prior to cull.

Loss of weight was one of the first observed signs of BSE and was seen significantly earlier in BSE-positive cases, although it was also present in inoculated animals that tested negative for BSE and in some control sheep (*P *< 0.05 by Wilcoxon test, see additional file 8: plots of the Kaplan-Meier estimate for weight loss). It frequently coincided with pruritic behaviour in BSE cases. Thus, limiting the clinical assessment to weighing and a scratch test can be done fairly quickly and may be an efficient way to screen sheep for BSE.

It was not investigated whether age at dosing affected the clinical presentation. Most of the sheep were challenged between four and six months of age, and the number of animals challenged at an older (nine months in AHQ/AHQ sheep) or younger age (possibly in the perinatal period in the two "naturally" infected animals) was too small to allow any statistical analysis. A separate study conducted in the UK where sheep were challenged with BSE at various ages has not found any obvious link between age of challenge, incubation period and type of clinical sign [17].

The potential for discriminating TSE strains by the clinical presentation was demonstrated in a mouse model where different prion strains produced different behavioural changes [30]. The authors hypothesised that this may also apply to larger animals although the range of tests that can be performed on sheep and cattle is obviously limited by the size and cooperation of the animals. BSE in cattle (C-type) is a neurological disease with a fairly uniform clinical presentation characterised by changes in behaviour, sensation and locomotion [31] and thought to be caused by a uniform strain based on a similarly uniform distribution and severity of vacuolar changes in cattle [32-34] and mice [35,36]. If strains predominantly dictate the clinical presentation, infection of sheep with the BSE agent should equally result in a relatively uniform clinical disease, regardless of breed and genotype, since the pathological phenotype, including the intracellular truncation pattern, remains constant when BSE is transmitted to sheep with different genotypes and by different routes [37,38]. Our results show that BSE in sheep indeed presents with a relatively uniform clinical disease with pruritus as the predominant and initial sign, accompanied by behavioural and later locomotor changes as the disease progresses, and only rarely deficits of the menace response. The two ARQ/ARQ Suffolk sheep inoculated intracerebrally displayed pruritus in a similar fashion to the BSE cases infected orally or naturally. A temporal distribution in the display of clinical signs has also been shown in sheep scrapie, with an increased frequency of altered mental status, pruritus with wool loss, teeth grinding, head tremor, ataxia, loss of bodily condition and an absent menace response as the disease progresses [8,14,15,39]. Our findings suggest that the clinical presentation of BSE in sheep of the susceptible or affected genotypes is largely influenced by the strain rather than breed, PrP genotype or route of inoculation although the vast majority of BSE cases were orally challenged ARQ/ARQ Romney or Suffolk sheep. More cases in other breeds or genotypes including orally inoculated VRQ/VRQ sheep need to be studied. Natural infection, although only described in two cases in an experimental flock, did present with similar clinical signs and incubation periods. No other classical scrapie strain has yet been found in sheep with similar uniform neuropathological, molecular and biological characteristics as BSE. Clinical cases of atypical scrapie, which may be caused by a single strain based on the neuropathological and molecular characteristics [40], display predominantly behavioural and locomotor abnormalities [10,11,41] but only very limited cases have been studied so far. Well-characterised scrapie isolates, such as SSPB/1 [42], produce a disease with variable clinical presentation in which pruritus and ataxia are displayed inconsistently in sheep of different breeds and genotypes inoculated subcutaneously [21]. The variation seen in the neuroanatomical distribution of vacuolar changes and PrP^d ^accumulation in the brain of sheep with scrapie is believed to be affected not only by the scrapie strain but also by sheep breed and host genotype [16,43,44]. Differentiation of ovine BSE from scrapie may not be possible based on clinical signs but our findings suggest that a scrapie suspect without signs of pruritus, such as the majority of atypical scrapie cases [10,11,41], is unlikely to have BSE.

## Conclusion

Oral BSE infection of Romney and Suffolk ARQ/ARQ sheep causes a uniform disease with early pruritus followed by behavioural changes and movement disorders as the disease progresses. Identical findings in a limited number of additional BSE cases described here suggest that the clinical features are independent of breed, genotype or route of inoculation. It is hypothesised that the clinical presentation in sheep is predominantly influenced by the TSE strain. Screening susceptible sheep for BSE may be done efficiently by regular monitoring of their weights and testing them for a scratch response.

## Methods

All procedures were approved under the Animal (Scientific Procedures) Act 1986. Each dose group was housed separately in medium security accommodations. The sheep were fed hay and straw *ad libitum *and a ration of feed concentrate free from animal protein/meat and bone meal, mixed with minerals.

The 139 sheep studied were sourced from scrapie-free flocks, which comprised clinically healthy castrated male or female Romney (n = 71), Suffolk (n = 59), Poll Dorset (n = 3) and Cheviot sheep (n = 6) of various genotypes [ARQ/ARQ (n = 59), ARQ/ARR (n = 35), ARR/ARR (n = 39), AHQ/AHQ (n = 4) and VRQ/VRQ (n = 2)]. The sheep were from four major BSE studies, an oral BSE pathogenesis study (n = 109) [22,45], a sheep to sheep BSE passage study (n = 13), a BSE research flock study (n = 15) [46,47] and a BSE tissue production study (n = 2).

### Oral BSE pathogenesis study

The experimental design has been published in detail for the Romney sheep study [22,45]. Briefly, 60 Romney and 56 Suffolk sheep of PrP genotypes ARQ/ARQ, ARQ/ARR or ARR/ARR were orally inoculated with 5 g of brainstem homogenate from histopathologically confirmed bovine BSE cases at 4–6 months of age. Control groups comprised a total of 15 and ten undosed Romney and Suffolk sheep, respectively, of the same genotypes. Sheep of the ARQ/ARQ genotype were culled at 4–6 month intervals from four to 22 mpi regardless of their clinical status; one group of four animals was kept alive until the development of clinical signs. Sheep of the ARQ/ARR (n = 18) and ARR/ARR (n = 17) genotypes were culled at 12-month intervals from 10 mpi to 46 mpi; the remaining animals were kept alive until the development of clinical disease. Another two ARR/ARR sheep were culled at 100–101 mpi for a bioassay in transgenic mice. One control sheep was culled at each timed cull of dosed animals.

Reported are the findings in 109 sheep, which included 61 Romney (19 ARQ/ARQ, 21 ARQ/ARR and ARR/ARR respectively) and 48 Suffolk sheep (17 ARQ/ARQ, 13 ARR/ARQ and 18 ARR/ARR). The majority (50 Romney and 43 Suffolk sheep) was challenged with BSE.

### Sheep to sheep passage study

Single oral doses of 0.0005 to 5 g (ten fold increase) brain homogenate from BSE-affected sheep provided by the oral BSE pathogenesis study were administered orally to sheep of the same or different breed and genotype at approximately six months of age to assess if the strain characteristics would change upon this second passage of BSE in sheep. For the purpose of this paper, we analysed the clinical findings in 13 sheep (four Romney, five Suffolk, three Poll Dorset with ARQ/ARQ genotype and one Romney with ARQ/ARR genotype), orally dosed with 5 g (n = 7), 0.5 g (n = 5) or 0.05 g (n = 1).

### BSE research flock

Sheep dosed orally with 5 g of bovine BSE brain were bred under strict biosecurity measures and mixed with undosed sheep to assess whether BSE can be transmitted naturally [46,47]. Animals were culled when they developed clinical disease.

Of the 12 sheep (four Suffolk and five Romney ARQ/ARQ sheep and three Cheviots with genotype AHQ/AHQ [n = 1] and VRQ/VRQ [n = 2], respectively) used for the analysis, ten were dosed ewes (age at dosing varied from four to nine months of age), and two were the offspring of dosed ewes, which included a Suffolk wether culled at 21 months of age [46] and a Romney ewe culled at 24 months of age.

### BSE tissue production study

Four ARQ/ARQ Suffolk sheep were inoculated intracerebrally with 1 ml of sterile bovine BSE brainstem (as a 10% homogenate in physiological saline solution) at eight months of age under general anaesthesia. Only two sheep had developed disease at the time of writing (April 2008, 38 mpi).

### Postmortem diagnosis

Diagnosis was made by immunohistochemical examination of the brain and – in case of a negative finding – lymphoreticular tissue, such as palatine tonsil, spleen, Peyer's patches of the distal ileum and various lymph nodes [22]. Western immunoblot examination of the caudal medulla ("VLA hybrid technique" [48]) was performed in animals negative by immunohistochemistry. The postmortem test results were used as the "gold standard" for this study; i.e. an animal that did not present with disease-associated prion protein (PrP^d^) in any of the examined tissues was considered as BSE negative, even if it was dosed.

### Clinical methods

For the oral pathogenesis study, clinical monitoring consisted of neurological examinations every three to four months (from 13 mpi), weekly behavioural group observations for about 30 minutes (from 14 mpi), and quarterly six-hour group behavioural studies (from 12–62 mpi in Romney sheep and 13–40 mpi in Suffolk sheep). Body weights were recorded monthly. Any deviation from normal was also recorded during daily or periodical husbandry procedures, such as feeding, cleaning of the pens, and shearing. Any deviation from normal usually resulted in more frequent neurological examinations and weighing sessions to determine clinical progression and the animals' fitness' to remain on the study (clinical end-point). The clinical methods were similar for the sheep to sheep passage study, apart from the exclusion of six-hour behavioural studies. The sheep from the BSE research flock and the BSE tissue production study were only examined once prior to cull.

The neurological examination [49] included gait assessment, testing of cranial nerve reflexes, and an evaluation of the scratch test response. For the scratch test, the animals were lightly restrained and scratched systematically along the dorsum. The scratch test was positive (positive scratch response) if the animal repeatedly displayed obvious stereotypical behaviour, such as nibbling movements of the lips or lip licking ("nibble reflex") or rhythmical head or body movements. An inconclusive scratch test result, such as occasional chewing movements or inconsistent stereotypical responses was considered "negative" for the purpose of this paper.

The clinical signs assessed at weekly behavioural observations included pruritic behaviour, such as scratching, rubbing on objects or nibbling at body parts. If animals displayed a distinct and repeatable stereotypical behaviour such as lip licking or smacking while rubbing parts of their body, this was recorded as a "self-induced scratch response". Other clinical signs noted included bruxism, snorting (voluntary expulsion of air through the nostrils), sneezing, yawning or flehmen, all of which have been observed in cattle with BSE [50]. Comments were also made on any deviation from normal sheep behaviour within the group (e.g. standing separate from the others) and when the observer entered the pen at the end of the observation period. The signs assessed during six-hour behavioural studies were similar although the pen was not entered at the end of these observations.

All examinations and observations were carried out according to a standard protocol originally set up by a certified veterinary neurologist (CBB). Each sign was defined, and the examiners/observers were trained to make the assessments as uniform as possible.

To document the clinical presentation and disease progression the incubation times were divided into four-week intervals and all signs displayed within each interval starting from the cull date were taken into consideration. Rubbing/scratching, nibbling and bruxism were graded from 0–4 (0 = never observed in a four-week interval, 1 = observed once, 2 = observed for up to 50% of the behavioural observations, 3 = observed for more than 50% of the behavioural observations). Weight loss was graded from 0–4 (0 = weight gain, 1 = weight remained the same compared to last weighing, 2 = weight loss of 5% or less of the previous body weight, 3 = weight loss between 5 and 10%, 4 = weight loss of more than 10%). The presence of other clinical signs was recorded as either "no" (= 0) or "yes" (= 1).

Behavioural changes included dull or nervous behaviour (not seen previously) and separation from the group. Skin lesions included excoriation, papules, lichenification or oedema, which have been observed in cases of scrapie [14,51]. Fleece changes included strips of wool hanging from the fleece or wool discolouration on the dorsum, shoulder or flank, which may have been the result of pruritus. Bruxism observed during the examination was distinguished from bruxism during passive behavioural observations because stress and handling may provoke teeth grinding even in healthy sheep (T Konold, unpublished observation).

Whether a given clinical sign was present in a sheep in a specific four-week interval could not always be assessed. For example weight loss could not be assessed if the animal had recently been shorn, nervous behaviour during handling could not be classified as "changed behaviour" if the sheep had not been handled previously. In addition, some clinical assessments were not possible during a foot and mouth disease epidemic in 2001 when access to the sheep accommodation was restricted to animal husbandry staff only.

As tremor in scrapie-affected sheep may only be evident when the animal is managed or driven [14,15] the assessment of tremor was only considered to be reliable when assessed at neurological examinations; the absence of this sign on passive observations was regarded as "not assessed" because it might have been displayed if the animal was stressed.

Clinical disease was suspected if sheep displayed a positive scratch test.

### Statistical analysis

The software packages SAS/STAT for Windows, version 9.1 (SAS International, Heidelberg, Germany), and Statistica, version 8 (StatSoft Inc., Tulsa, USA), were used. Individual clinical signs between positive and negative cases were compared by Fisher's exact test. Survival analysis (log-rank test and Wilcoxon test if the assumption of proportional hazards was not fulfilled) was used for clinical signs observed during the period of clinical monitoring by comparing the times of the first appearance of a particular clinical sign in positive and negative cases. For this analysis, only the presence or absence of a clinical sign was used, and the grading system for the signs rubbing/scratching, nibbling, bruxism at observation and weight loss was ignored.

A *P*-value of less than 0.05 was considered to be statistically significant.

## Authors' contributions

CBB and TK set up the clinical methods and definitions. CBB, TK, RT, AD, PH, GD, PH and GB were actively involved in the clinical assessment of the animals. The data was analysed by TK, AVD, GB and CBB. SJB with the aid of GD and PH managed the Defra-funded sheep studies, whilst MMS managed the EC-funded sheep study. The neuropathological examinations were carried out by MJ and MMS. MJC was responsible for the Western immunoblot examinations. All authors read and approved the final manuscript.

## Supplementary Material

Additional file 1 "Scratch response in orally challenged VRQ/VRQ sheep". Cheviot ewe (VRQ/VRQ, animal T1714) at 51 months after oral challenge with 5 g of BSE brainstem homogenate. This ewe responds to scratching of the dorsum initially with raising of the head and lowering of the hind quarters but eventually displays a scratch response (nibbling). Click here for file

Additional file 2 "Scratch response in an intracerebrally inoculated ARQ/ARQ sheep". Suffolk wether (ARQ/ARQ, animal 05–1015) at 18 months after intracerebral inoculation with BSE brainstem homogenate. Scratching of the dorsum elicits a scratch response with excessive bending of the neck to the right/upwards. Click here for file

Additional file 3 "Self-induced scratch response". Romney ewe (ARQ/ARQ, animal SM1406) at 37 month after oral challenge with 5 g of BSE brainstem homogenate. Rubbing of the dorsum on the water trough induces a scratch response (nibbling). Click here for file

Additional file 4 "Self-nibbling". Group of four Suffolk sheep (ARQ/ARQ) at 17 months after intracerebral inoculation with BSE brainstem homogenate. Two sheep (wether 05–1015, lying next to black plastic drum, and ewe 05–1022, lying down in front of the camera) display pruritic behaviour, such as nibbling of the left flank and/or hind limb. Around the same time, both sheep also displayed a scratch response when the dorsum was scratched (see animal 05–1015 in additional file 2). The other two sheep were still clinically healthy 20 months later. Click here for file

Additional file 5 "Scratch response in orally challenged ARQ/ARQ sheep". Romney ewe (ARQ/ARQ, animal SR5169) at 25 months after oral challenge with 0.5 g of BSE brainstem homogenate. This sheep displays a scratch response (nibbling) when the dorsum at the level of the thoracic spinal column is scratched. Even mere touching of the sheep's dorsum further caudal elicits a brief 'nibble' as can be seen at the end of the clip. Click here for file

Additional file 6 "Scratch response in an orally challenged AHQ/AHQ sheep". Cheviot ewe (AHQ/AHQ, animal 06–1069) at 18 months after oral challenge with 5 g of BSE brainstem homogenate. This ewe responds to scratching with raising of the head only when restrained during the scratch test, but displays rhythmical lip and body movements when scratched whilst standing freely in the pen. Click here for file

Additional file 7 "Ataxia with mild proprioceptive deficits in orally challenged ARQ/ARQ sheep". Romney ewe (ARQ/ARQ, animal SR5169) at 25 months after oral challenge with 0.5 g of BSE brainstem homogenate (see additional file 5). Whilst testing the proprioceptive positioning response of the right hind limb the ewe is briefly knuckling over its left hind fetlock when it tries to correct the position of the right hind limb. It displays a 'swaying gait' with occasional abnormal placement of the hind limbs. (Note the very slight loss of balance with leaning to the left when the ewe approaches the end of the corridor a second time.) Click here for file

Additional file 8 Plots of Kaplan-Meier estimates for the first appearance of each clinical sign in negative and positive BSE cases. Click here for file

## References

[B1] Bruce ME, Will RG, Ironside JW, McConnell I, Drummond D, Suttie A, McCardle L, Chree A, Hope J, Birkett C, Cousens S, Fraser H, Bostock CJ (1997). Transmissions to mice indicate that 'new variant' CJD is caused by the BSE agent. Nature.

[B2] Foster JD, Parnham DW, Hunter N, Bruce M (2001). Distribution of the prion protein in sheep terminally affected with BSE following experimental oral transmission. J Gen Virol.

[B3] Baylis M (2002). The BSE-susceptible proportion of UK sheep. Vet Rec.

[B4] Houston F, Goldmann W, Chong A, Jeffrey M, González L, Foster J, Parnham D, Hunter N (2003). Prion diseases: BSE in sheep bred for resistance to infection. Nature.

[B5] Goldmann W, Houston F, Stewart P, Perucchini M, Foster J, Hunter N (2006). Ovine prion protein variant A136R154L168Q171 increases resistance to experimental challenge with bovine spongiform encephalopathy agent. J Gen Virol.

[B6] Vaccari G, D'Agostino C, Nonno R, Rosone F, Conte M, Di Bari MA, Chiappini B, Esposito E, De Grossi L, Giordani F, Marcon S, Morelli L, Borroni R, Agrimi U (2007). Prion protein alleles showing a protective effect on the susceptibility of sheep to scrapie and bovine spongiform encephalopathy. J Virol.

[B7] Sigurdsson B (1954). Rida, a chronic encephalitis of sheep: With general remarks on infections which develop slowly and some of their special characteristics. Br Vet J.

[B8] Parry HP (1957). Scrapie and related myopathies in sheep. Vet Rec.

[B9] Joubert L, Lapras M, Gastellu J, Prave M, Laurent D (1972). Un foyer de tremblante du mouton en Provence. Bull Soc Sci Vet Med Comp.

[B10] Benestad SL, Sarradin P, Thu B, Schönheit J, Tranulis MA, Bratberg B (2003). Cases of scrapie with unusual features in Norway and designation of a new type, Nor98. Vet Rec.

[B11] Konold T, Davis A, Bone G, Bracegirdle J, Everitt S, Chaplin M, Saunders GC, Cawthraw S, Simmons MM (2007). Clinical findings in two cases of atypical scrapie in sheep: a case report. BMC Vet Res.

[B12] Pattison IH, Millson GC (1961). Scrapie produced experimentally in goats with special reference to the clinical syndrome. J Comp Pathol.

[B13] Morales R, Abid K, Soto C (2007). The prion strain phenomenon: Molecular basis and unprecedented features. Biochim Biophys Acta.

[B14] Healy AM, Weavers E, McElroy M, Gomez-Parada M, Collins JD, O'Doherty E, Sweeney T, Doherty ML (2003). The clinical neurology of scrapie in Irish sheep. J Vet Intern Med.

[B15] Vargas F, Lujan L, Bolea R, Monleon E, Martin-Burriel I, Fernandez A, De Blas I, Badiola JJ (2006). Detection and clinical evolution of scrapie in sheep by 3rd eyelid biopsy. J Vet Intern Med.

[B16] Begara-McGorum I, González L, Simmons M, Hunter N, Houston F, Jeffrey M (2002). Vacuolar lesion profile in sheep scrapie: factors influencing its variation and relationship to disease-specific PrP accumulation. J Comp Pathol.

[B17] Foster JD, Parnham D, Chong A, Goldmann W, Hunter N (2001). Clinical signs, histopathology and genetics of experimental transmission of BSE and natural scrapie to sheep and goats. Vet Rec.

[B18] Foster JD, Hope J, Fraser H (1993). Transmission of bovine spongiform encephalopathy to sheep and goats. Vet Rec.

[B19] Baron TGM, Madec JY, Calavas D, Richard Y, Barillet F (2000). Comparison of French natural scrapie isolates with bovine spongiform encephalopathy and experimental scrapie infected sheep. Neurosci Lett.

[B20] Thuring CMA, van Keulen LJM, Langeveld JPM, Vromans MEW, van Zijderveld FG, Sweeney T (2005). Immunohistochemical distinction between preclinical bovine spongiform encephalopathy and scrapie infection in sheep. J Comp Pathol.

[B21] Houston EF, Gravenor MB (2003). Clinical signs in sheep experimentally infected with scrapie and BSE. Vet Rec.

[B22] Jeffrey M, Ryder S, Martin S, Hawkins SA, Terry L, Berthelin-Baker C, Bellworthy SJ (2001). Oral inoculation of sheep with the agent of bovine spongiform encephalopathy (BSE). 1. Onset and distribution of disease-specific PrP accumulation in brain and viscera. J Comp Pathol.

[B23] Ersdal C, Ulvund MJ, Espenes A, Benestad SL, Sarradin P, Landsverk T (2005). Mapping PrPSc propagation in experimental and natural scrapie in sheep with different PrP genotypes. Vet Pathol.

[B24] Appleyard B, Bailie H (1984). Parasitic skin diseases of sheep. In Pract.

[B25] Sargison N (1995). Differential diagnosis and treatment of sheep scab. In Pract.

[B26] March PA (1998). Seizures: classification, etiologies, and pathophysiology. Clin Tech Small Anim Pract.

[B27] Mayhew IG (1989). Large Animal Neurology A handbook for veterinary clinicians.

[B28] Espenes A, Press CM, Landsverk T, Tranulis MA, Aleksandersen M, Gunnes G, Benestad SL, Fuglestveit R, Ulvund MJ (2006). Detection of PrP(Sc) in rectal biopsy and necropsy samples from sheep with experimental scrapie. J Comp Pathol.

[B29] Kim FB, Jackson RE, Gordon GDH, Cockram MS (1994). Resting behaviour of sheep in a slaughterhouse lairage. Appl Anim Behav Sci.

[B30] Dell'Omo G, Vannoni E, Vyssotski AL, Di Bari MA, Nonno R, Agrimi U, Lipp HP (2002). Early behavioural changes in mice infected with BSE and scrapie: automated home cage monitoring reveals prion strain differences. Eur J Neurosci.

[B31] Schicker E, Braun U, Hörnlimann B, Konold T, Hörnlimann B, Riesner D and Kretzschmar H (2006). Clinical findings in bovine spongiform encephalopathy. Prions in humans and animals.

[B32] Simmons MM, Harris P, Jeffrey M, Meek SC, Blamire IWH, Wells GAH (1996). BSE in Great Britain: Consistency of the neurohistopathological findings in two random annual samples of clinically suspect cases. Vet Rec.

[B33] Breslin P, McElroy M, Bassett H, Markey B (2006). Vacuolar lesion profile of BSE in the Republic of Ireland. Vet Rec.

[B34] Gubler E, Hilbe M, Ehrensperger F (2007). Lesion profiles and gliosis in the brainstem of 135 Swiss cows with bovine spongiform encephalopathy (BSE). Schweiz Arch Tierheilkd.

[B35] Bruce ME, Boyle A, Cousens S, McConnell I, Foster J, Goldmann W, Fraser H (2002). Strain characterization of natural sheep scrapie and comparison with BSE. J Gen Virol.

[B36] Green R, Horrocks C, Wilkinson A, Hawkins SAC, Ryder SJ (2005). Primary isolation of encephalopathy agent based on a review the bovine spongiform in mice: Agent definition of 150 transmissions. J Comp Pathol.

[B37] González L, Martin S, Houston FE, Hunter N, Reid HW, Bellworthy SJ, Jeffrey M (2005). Phenotype of disease-associated PrP accumulation in the brain of bovine spongiform encephalopathy experimentally infected sheep. J Gen Virol.

[B38] Martin S, González L, Chong A, Houston FE, Hunter N, Jeffrey M (2005). Immunohistochemical characteristics of disease-associated PrP are not altered by host genotype or route of inoculation following infection of sheep with bovine spongiform encephalopathy. J Gen Virol.

[B39] Sharpe A, McElroy M, Bassett H, Sweeney T (2006). Clinical and pathological features of experimental scrapie in Irish Blackface Mountain sheep. Res Vet Sci.

[B40] EFSA (2005). Opinion of the scientific panel on biological hazards on the request from the European Commission on classification of atypical transmissible spongiform encephalopathy (TSE) cases in small ruminants. EFSA J.

[B41] Simmons MM, Konold T, Simmons HA, Spencer YI, Lockey R, Spiropoulos J, Everitt S, Clifford D (2007). Experimental transmission of atypical scrapie to sheep. BMC Vet Res.

[B42] Dickinson AG, Kimberlin RH (1976). Scrapie in sheep and goats. Slow virus diseases of animals and man.

[B43] Gonzalez L, Martin S, Begara-McGorum I, Hunter N, Houston F, Simmons M, Jeffrey M (2002). Effects of agent strain and host genotype on PrP accumulation in the brain of sheep naturally and experimentally affected with scrapie. J Comp Pathol.

[B44] Spiropoulos J, Casalone C, Caramelli M, Simmons MM (2007). Immunohistochemistry for PrPSc in natural scrapie reveals patterns which are associated with the PrP genotype. Neuropathol Appl Neurobiol.

[B45] Bellworthy SJ, Hawkins SAC, Green RB, Blamire I, Dexter G, Dexter I, Lockey R, Jeffrey M, Ryder S, Berthelin-Baker C, Simmons MM (2005). Tissue distribution of bovine spongiform encephalopathy infectivity in Romney sheep up to the onset of clinical disease after oral challenge. Vet Rec.

[B46] Bellworthy SJ, Dexter G, Stack M, Chaplin M, Hawkins SAC, Simmons MM, Jeffrey M, Martin S, González L, Hill P (2005). Natural transmission of BSE between sheep within an experimental flock. Vet Rec.

[B47] Bellworthy SJ, Dexter G, Stack M, Chaplin M, Hawkins SAC, Simmons MM, Jeffrey M, Martin S, González L, Martin S, Hill P (2008). Oral transmission of BSE to VRQ/VRQ sheep in an experimental flock. Vet Rec.

[B48] Stack MJ, Chaplin MJ, Clark J (2002). Differentiation of prion protein glycoforms from naturally occurring sheep scrapie, sheep-passaged scrapie strains (CH1641 and SSBP1), bovine spongiform encephalopathy (BSE) cases and Romney and Cheviot breed sheep experimentally inoculated with BSE using two monoclonal antibodies. Acta Neuropathol.

[B49] Wells GAH, Hawkins SAC, Lehmann S and Grassi J (2004). Animal models of transmissible spongiform encephalopathies: Experimental infection, observation and tissue collection. Techniques in prion research.

[B50] Austin AR, Hawkins SAC, Kelay NS, Simmons MM, Bradley R and Marchant B (1994). New observations on the clinical signs of BSE and scrapie. Transmissible spongiform encephalopathies Proceedings of a consultation on BSE with the Scientific Veterinary Committee of the Commission of the European Communities: 14-15 September 1993; Brussels.

[B51] Ulvund MJ, Hörnlimann B, Riesner D and Kretzschmar H (2006). Clinical findings in scrapie. Prions in humans and animals.

