# Supplementary material for: Pruritus is a common feature in sheep infected with the BSE agent
Source: BMC Vet Res. 2008 Apr 29;4:16. doi: 10.1186/1746-6148-4-16 (PMC2390527; doi:10.1186/1746-6148-4-16)

# Kaplan-Meier estimates for the first appearance of each clinical sign in BSE-positive and negative sheep

# 1. Change in behaviour


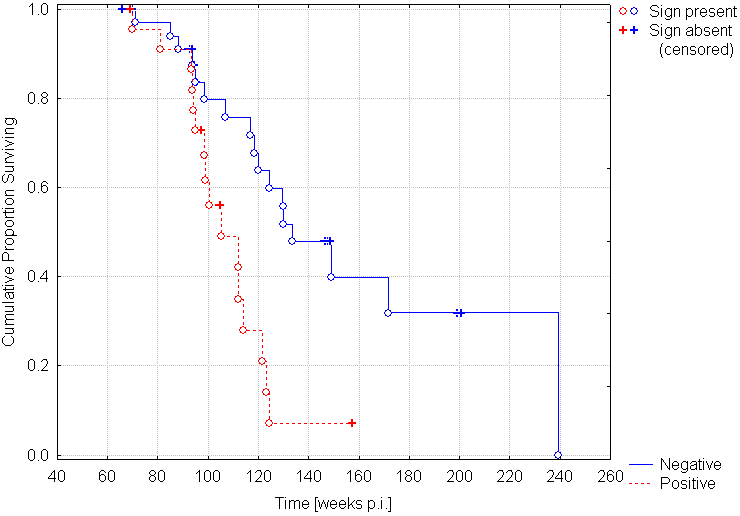


# 2. Bruxism at neurological examination when free


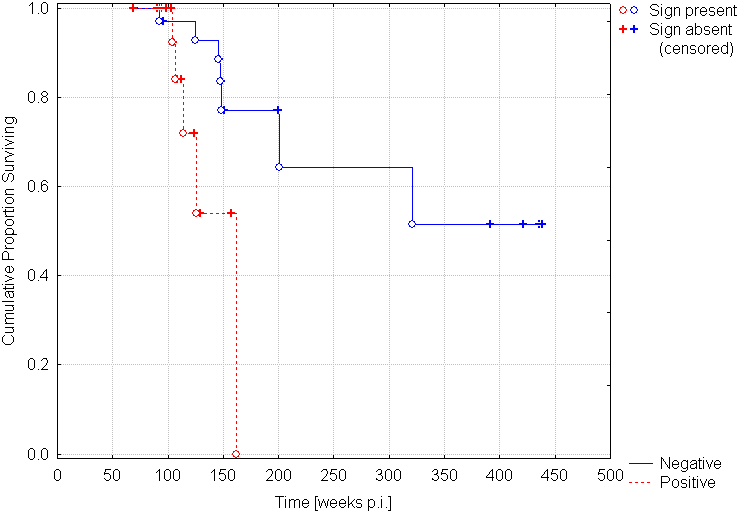


**3. Bruxism at neurological examination when handled**


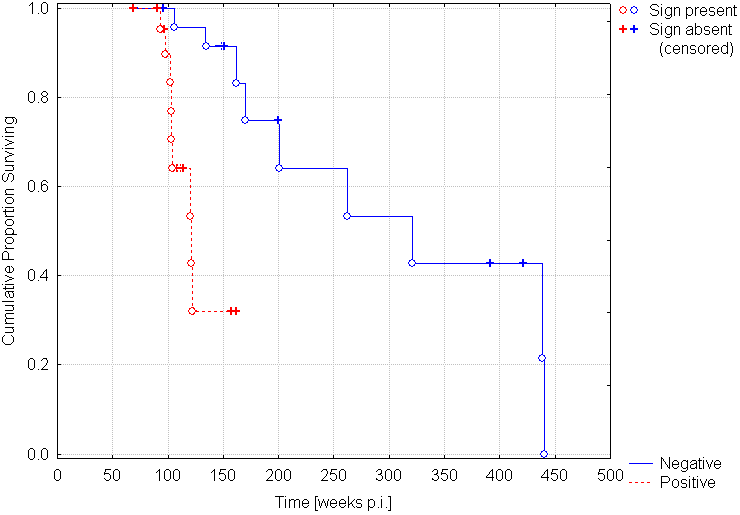


# 4. Bruxism at passive observation


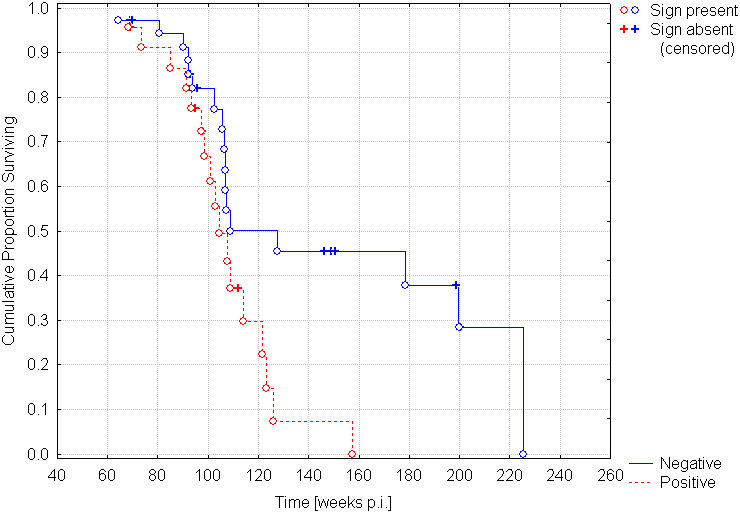


# 5. Rubbing or scratching of body parts at passive observation


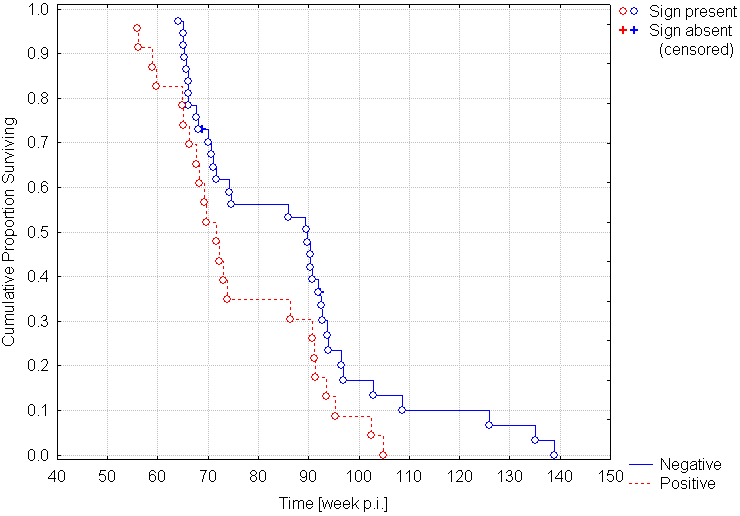


# 6. Nibbling of body parts at passive observation


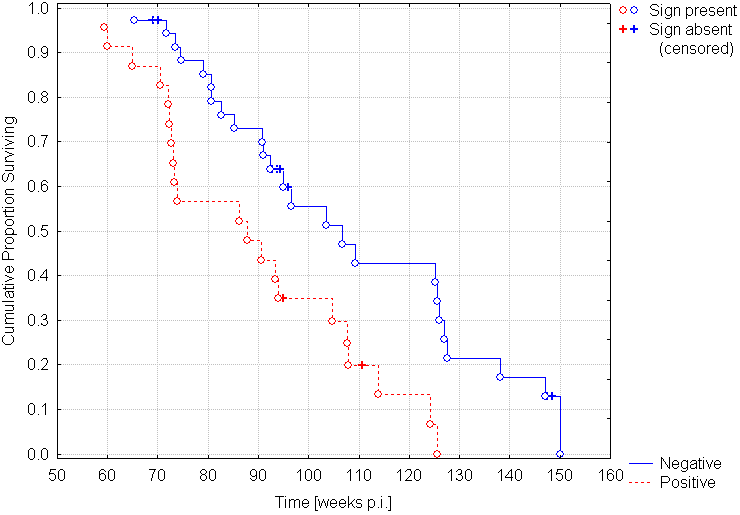


# 7. Positive scratch test


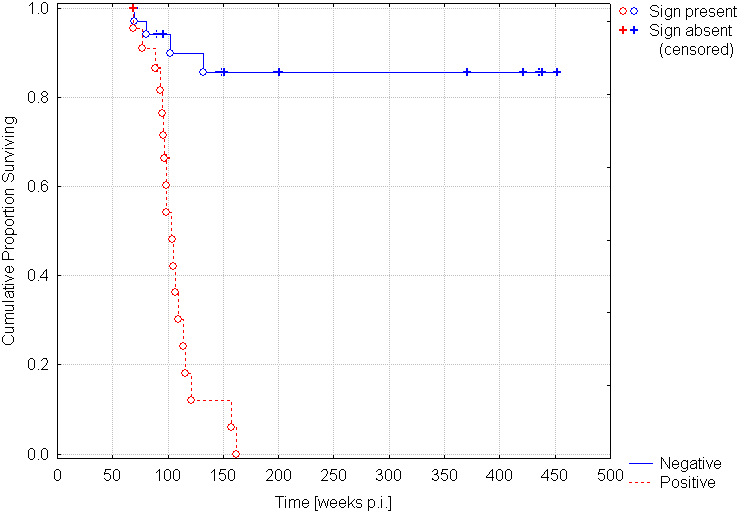


# 8. Fleece changes


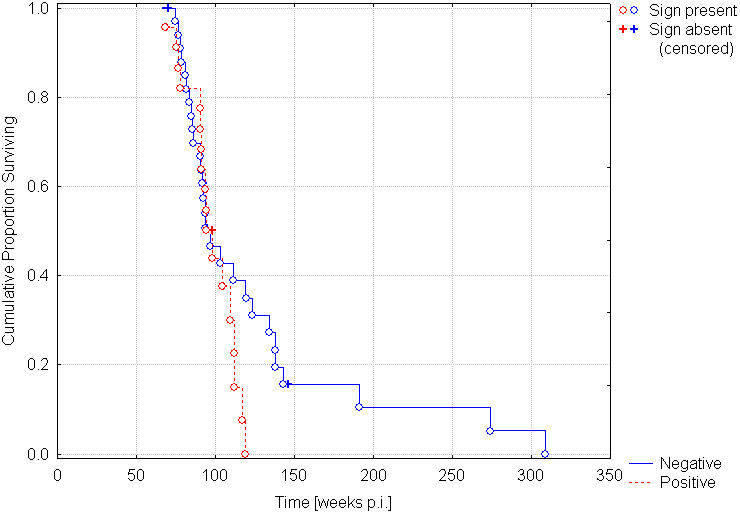


# 9. Alopecia


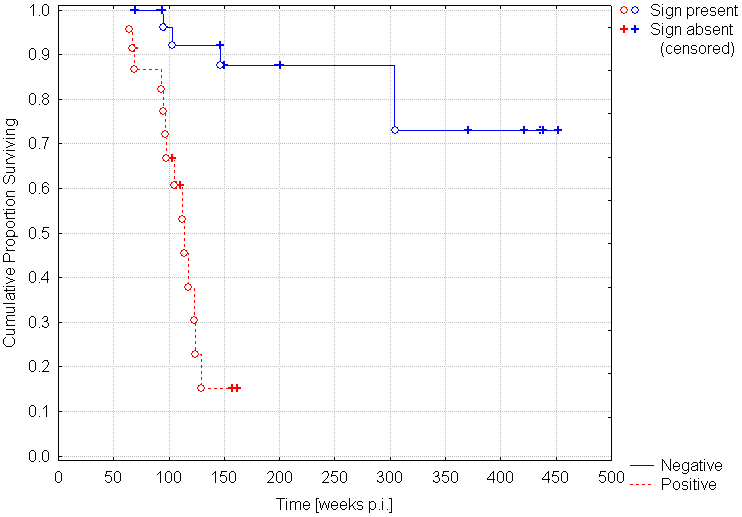


# 10. Skin lesions


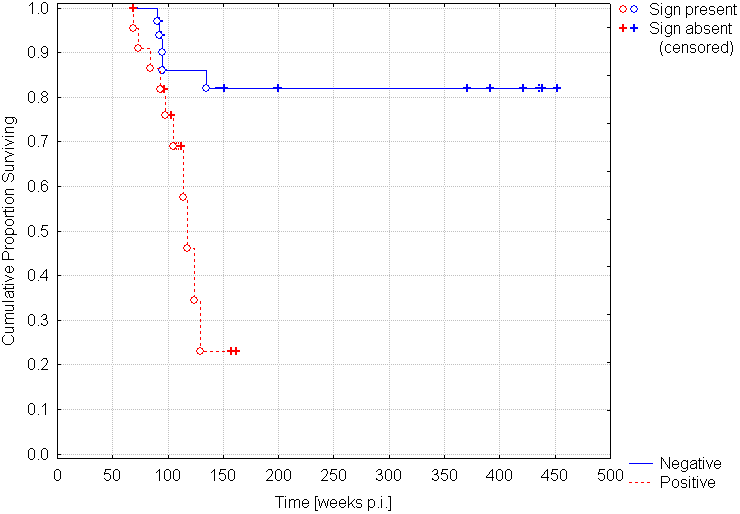


# 11. Self-induced nibble reflex


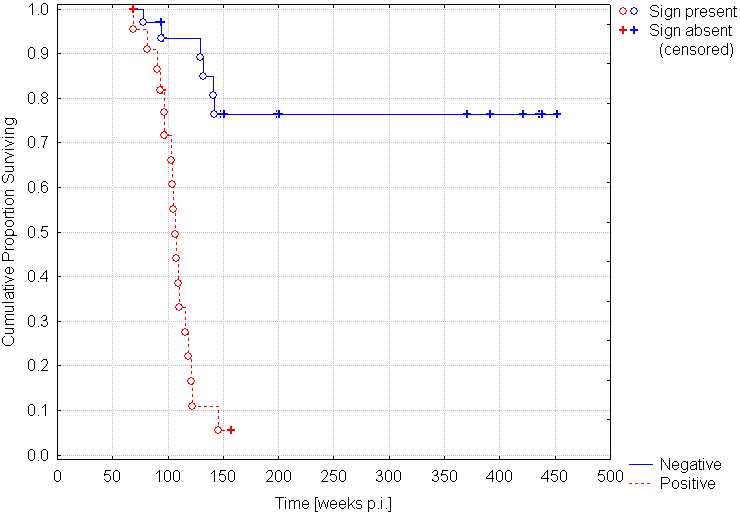


# 12. Tremor


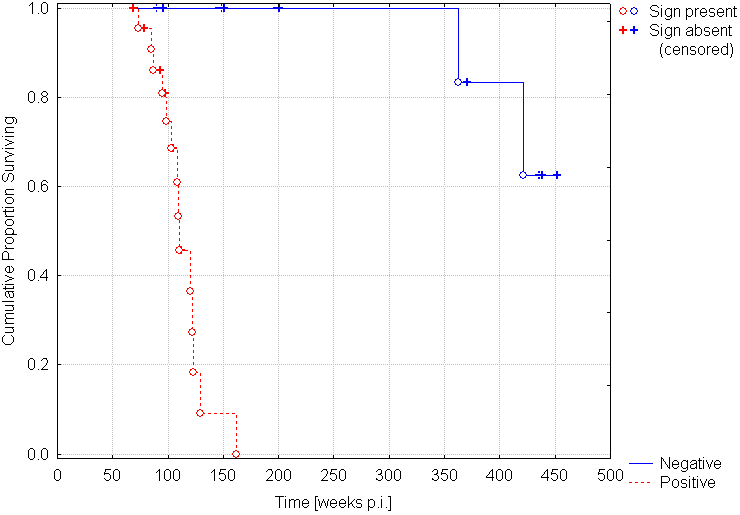


# 13. Ataxia

#
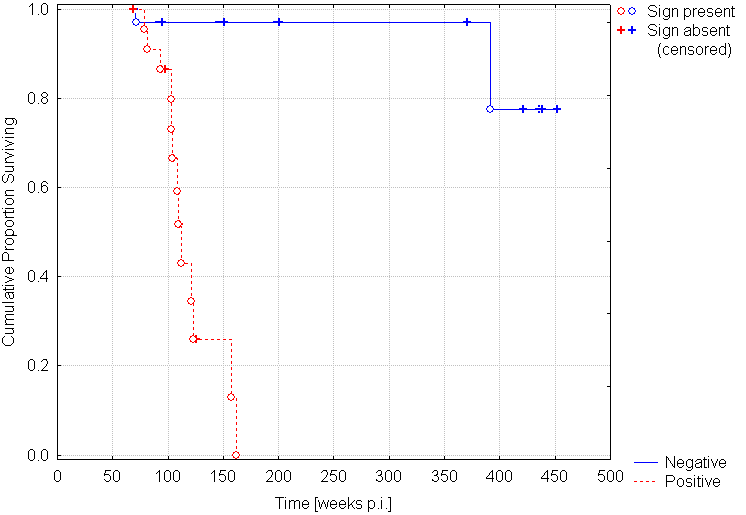


# 14. Loss of weight


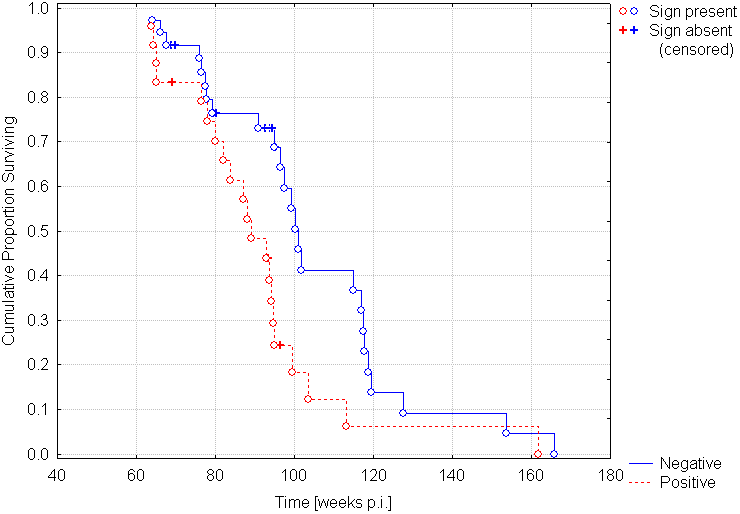


# 15. Loss of body condition


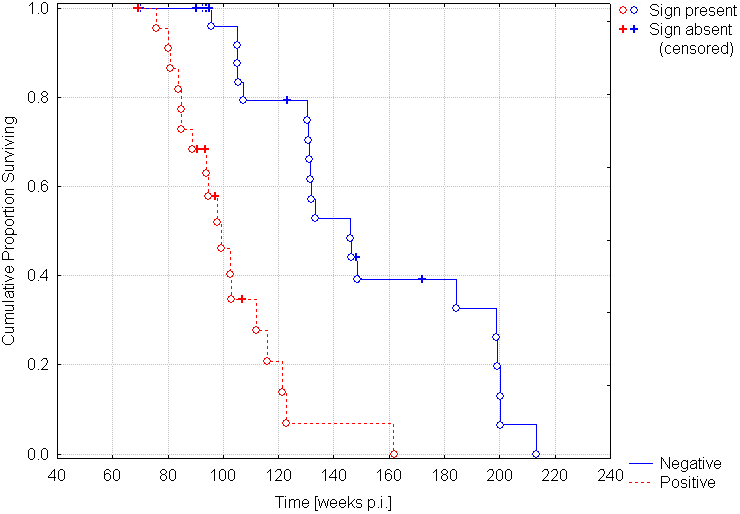

Supplement: Additional file 8 — Plots of Kaplan-Meier estimates for the first appearance of each clinical sign in negative and positive BSE cases. [file 1746-6148-4-16-S8.doc]
